# Artichoke By-Product Extracts as a Viable Alternative for Shelf-Life Extension of Breadsticks

**DOI:** 10.3390/foods13162639

**Published:** 2024-08-22

**Authors:** Michela Cannas, Paola Conte, Antonio Piga, Alessandra Del Caro

**Affiliations:** Department of Agricultural Sciences, Università degli Studi di Sassari, Viale Italia 39/A, 07100 Sassari, Italy; mcannas@uniss.it (M.C.); pigaa@uniss.it (A.P.); delcaro@uniss.it (A.D.C.)

**Keywords:** artichoke by-product extracts, breadsticks, fortification, hydroalcoholic extracts, antioxidant capacity, estimated shelf-life, OXITEST

## Abstract

The upcycling of agricultural by-products and the extension of the shelf-life of staple foods represent crucial strategies for mitigating the consequences of food losses and enhancing the competitiveness of the agri-food industry, thus facilitating the attainment of higher financial revenues. This is particularly relevant for global artichoke cultivation, where 60–80% of its biomass is discarded annually. The present study investigated the potential of using non-stabilized polyphenol-rich extracts from the main artichoke by-products (bracts, leaves, and stems) to fortify and extend the shelf-life of breadsticks. The incorporation of hydroalcoholic extracts at two addition levels (1000–2000 ppm) resulted in an increased antioxidant capacity and oxidative stability of fortified breadsticks. Rheological tests revealed that the fortification did not affect the dough’s workability, with the exception of the leaf extract. While a slight deterioration in texture was observed, the shelf-life of breadsticks was significantly extended, particularly at the highest levels of addition, without any visible alteration in their appearance. The stem extract demonstrated the most promising outcomes, exhibiting a maximum increase of 69% in antioxidant capacity (DPPH) and an extension of the estimated shelf-life by 62% in the resulting breadsticks, prompting the potential for utilizing them to develop nutritious and healthy snacks with extended shelf-life.

## 1. Introduction

According to FAO estimates, one-third of the food produced for human consumption annually, which is equivalent to about 1.3 billion tons, is lost or wasted worldwide, along the entire supply chain, from agricultural production to final household consumption [1]. In recent years, one of the primary objectives has been to guarantee food security and environmental sustainability. This has entailed the production of safe and nutritious foods while reducing food losses, with the implementation of cost-effective solutions. The growing attention to this problem is reflected in the United Nations Sustainable Development Goals, which call for a reduction in per capita food waste by 50% by 2030 [2]. The fruit and vegetable sector suffers from a high incidence of food surplus and waste, and a food loss rate of about 22% from post-harvest to distribution is realistic [3]. A reduction in wastage, especially in industrialized countries, could be attained by intervening along the various stages of the supply chain, via different strategies, for example, the reuse of fruit and vegetable by-products, which are extremely rich in bioactive compounds, to obtain functional ingredients with high added value. In addition, these by-products, which are also replete with antioxidants, can be employed to delay lipid oxidation and extend the shelf-life of food products. The term “shelf-life” is not used to define the real life of the product; rather, it represents the period of time that ends when the product undergoes physical and sensory deterioration that cannot be tolerated by the consumer, which compromises its marketability [4]. In fact, a longer shelf-life should minimize domestic waste, considering the careless attitude to manage food provision of most consumers, and reduce the economic and environmental impacts of the distribution logistics [5]. Baked products such as breadsticks are distinguished by a low water activity, which gives them a long stability. However, they are prepared by including high amounts of lipids, such as unsaturated vegetable oils or animal fat, in their basic formulation. Consequently, lipid oxidation is one of the primary causes of the quality decay of breadsticks [6,7]. To counteract lipid oxidation and extend their shelf-life, antioxidants can be added to their formulation [6,7,8]. Furthermore, these cylindrical-shaped snacks are commonly eaten all over the world as appetizers due to their convenience, crunchiness, and taste. Consequently, they can be easily fortified with functional ingredients and employed as carriers of bioactive compounds to confer additional health benefits, given that they are staple foods and are consumed on a daily basis. Only a few studies concerning the impact of incorporating by-products or by-product extracts on the shelf-life and oxidative stability of breadsticks are available in the literature. Conte et al. (2021), investigating the effect of the incorporation of phenolic-rich extracts from olive leaves and olive mill wastewater into gluten-free breadstick formulations, observed an increase in the content of polyphenols and a significant extension of their shelf-life [7]. By replacing wheat flour with grape pomace powder, Bianchi et al. (2021) reported an improvement in the nutritional profile but a worsening in the oxidative stability of breadsticks [6]. In another work, breadsticks fortified with different amounts of brewer’s spent grain were found to be quite stable, in terms of both texture and water activity, during storage, and with an implemented protein and dietary fiber content [9]. To date, no research has been conducted to evaluate the effect of artichoke by-product fortification on the oxidation stability and shelf-life of breadsticks.

Indeed, the globe artichoke (*Cynara cardunculus* L. subsp. scolymus L.), consumed worldwide, represents an important agro-economic source for the Mediterranean Basin, which produces considerable amounts of wastage (60–80% of the total biomass). The main by-products derived from the processing industry are outer bracts, leaves, and stems, which, like the edible parts, contain a great variety of natural antioxidants, such as polyphenols, particularly phenolic acids and flavonoids [10,11,12]. In general, agri-food wastes are transformed into powders, flours, or, less frequently, extracts for incorporation into bakery products. This is carried out in order to improve the nutritional value of the products, particularly in terms of phenolic compound content and antioxidant capacity [13]. The most straightforward approach for enhancing the nutritional profile of staple foods is the use of powders. Indeed, the same authors recently conducted a study in which they successfully fortified breadsticks by adding increasing percentages (3% and 5%) of powdered by-products such as artichoke stems and bracts [14]. The incorporation of these by-products significantly increased the nutritional and textural properties as well as the antioxidant capacity of the breadsticks. This evidence substantiates the potential of artichoke by-products as a fortification strategy for this type of bakery product.

Nevertheless, the utilization of extracts may be of interest, particularly with regard to the prolongation of the shelf-life of baked goods. This is due to the fact that they are more concentrated than their initial matrices in antioxidants, which can be added in small amounts without significantly altering the appearance of the final product. Extracts are usually stabilized to protect the more labile bioactive compounds, through techniques such as spray drying and freeze drying, resulting in higher production costs [15]. In this context, the present study aimed to evaluate the feasibility of employing polyphenol-rich extracts derived from artichoke bract, stem, and leaf fractions to improve the antioxidant capacity and shelf-life of breadsticks. In order to minimize the economic impact and comply with the principles of sustainability, the three hydroalcoholic extracts (prepared with food-grade ethanol) were added directly to the formulations without any further processing. Different phytochemical species can be extracted from each by-product fraction [10], and consequently, distinct effects could be observed in the enriched product. To accomplish this, two different levels (1000 ppm and 2000 ppm) of each by-product extract were individually incorporated into the formulation. Firstly, the rheological properties and the polyphenol content of the enriched doughs were evaluated to assess both the effect of the extract addition on the workability of the dough and if there was any loss of polyphenols as a result of the baking process. In addition to the polyphenol content and antioxidant capacity, chemical–physical and structural properties of the breadsticks were measured to determine whether fortification could affect the quality of the final product. The shelf-life of breadstick samples was estimated by using OXITEST, which was found to be an excellent and innovative tool for estimating the shelf-life of baked goods in a short time [16].

## 2. Materials and Methods

### 2.1. Raw Materials

Breadstick ingredients (wheat flour, sunflower oil, fresh compressed yeast, and salt) were purchased from a local grocery store. “Spinoso sardo” artichoke (*Cynara scolymus* L.) by-product fractions (stems, leaves, and outer bracts), from the 2020 crop season, were supplied by the North Sardinian companies of the consortium “Carciofo Spinoso di Sardegna D.O.P.”.

### 2.2. By-Product Extract Preparation

The artichoke by-products were individually freeze-dried and finely milled and extracted in accordance with procedures optimized by the same authors for each fraction in a previous study [10]. This was performed with the objective of obtaining the highest polyphenol content present in each fraction. To this end, the leaf extract was obtained by macerating 1 g of freeze-dried leaves for 60 min at a temperature of 38 ± 2 °C using 20 mL of a 45% ethanol (Carlo Erba Reagents., Ltd., Milan, Italy) hydroalcoholic solution. The stems and bracts were subjected to ultrasound-assisted extraction (frequency of 40 kHz and power set at 144 W) using a hydroalcoholic solution (with a concentration of 42% ethanol for stems and 64% ethanol for bracts) at a ratio of 1/20 (*w*/*v*) for 10 and 41 min, respectively. The decision to proceed with ultrasonic-assisted extraction for the bracts and stems, rather than maceration, was made on the grounds that this approach still permitted a high level of polyphenol recovery while offering significant time savings. Subsequently, all three extracts were centrifuged for 10 min at 9000 rpm, filtered through cellulose acetate syringe filters (0.45 μm pore-size), and stored at −20 °C before use.

### 2.3. Total Polyphenol Content and Antioxidant Capacity of By-Product Extracts

The total polyphenol content of the extracts obtained from artichoke by-products (bracts, leaves, and stems) was determined following the Folin–Ciocalteu method, reported by Cannas et al. (2023) [10]. Specifically, 1 mL of extract was mixed with 7.5 mL of distilled water, and then 0.5 mL of Folin–Ciocalteau reagent (50%) (Carlo Erba Reagents, Ltd., Milan, Italy) and 1 mL of sodium carbonate (10%) (Carlo Erba Reagents, Ltd., Milan, Italy) were added. After 1 h in the dark at room temperature, the absorbance was measured in a spectrophotometer (Agilent, model Cary 3500, Cernusco, Milan, Italy) at a wavelength of 765 nm, and the results were expressed as mg of gallic acid (Carlo Erba Reagents, Ltd., Milan, Italy) equivalent (GAE) per 100 g of dry matter (d.m.).

Antioxidant capacity was determined using two different spectrophotometric methods (ABTS and DPPH) according to Cannas et al. (2023) [10]. In the DPPH assay, 70 μL of the extract was reacted in the dark with 2.03 mL of a DPPH–methanol solution (0.1 mM) (Sigma-Aldrich, Milan, Italy), having an initial absorbance of 1.0 ± 0.2 (obtained by correcting with methanol additions). After 30 min under constant stirring, the drop in DPPH absorbance was measured using a spectrophotometer set at a wavelength of 517 nm. In the ABTS assay, a solution was prepared by combining equal volumes of ABTS (7.4 mM) (Sigma-Aldrich, Milan, Italy) and potassium persulfate (2.6 mM) (Sigma-Aldrich, Milan, Italy), both prepared with phosphate buffer (Sigma-Aldrich, Milan, Italy) and left to react for at least 12 h in the dark at room temperature (20–22 °C). The resulting solution was then further diluted with phosphate buffer before use to obtain an initial absorbance of 1.0 ± 0.2 at 734 nm. Then, 40.8 μL of extract was added to 2 mL of ABTS working solution. The absorbance values at 734 nm were measured after 6 min of incubation at 22 °C in the dark. Concentration–response curves produced with standard Trolox (Sigma-Aldrich, Milan, Italy) solutions were used to calculate the antioxidant capacity, and the results were expressed for both assays as μmol of Trolox equivalent (TE) per 1 g of d.m. The assays were performed in duplicate.

### 2.4. Dough and Breadstick Preparation

The samples were fortified by individually adding the hydroalcoholic extracts of artichoke outer bracts (BE), leaves (LE), and stems (SE) to the base formulation, at two different addition levels: level 1 (1000 ppm) and level 2 (2000 ppm). These levels were selected based on data reported in the literature [7,17] and preliminary laboratory trials, which demonstrated that higher fortification levels negatively impact dough machinability, particularly during the forming stage.

The control formulation consisted of type 0 flour, 50% water, 10% sunflower oil, 3% compressed yeast, and 1.8% salt (% based on flour).

Samples for dough analysis were prepared in duplicate by kneading the ingredients provided in the formulation without the addition of yeast, using a mixer (KitchenAid Professional, Model 5KSM7990, St. Joseph, MI, USA) furnished with a dough hook, at speed 2 for 6 min. The resulting doughs were immediately frozen (−20 °C), lyophilized after 24 h, finely ground, and stored at −20 °C until analysis.

The breadsticks were prepared by first suspending the extracts, yeast, and salt in different aliquots of water (about 26 °C), which were then added to the flour and sunflower oil and kneaded (6 min, speed 2). The doughs obtained were subsequently laminated (Domino S.r.l., Model SFO600, Schio, Italy) to a final thickness of 0.3 cm, cut into 18 cm long sheets, and formed using a breadstick machine (Italpan, Model AFP/GR15, Schio, Italy) equipped with 1 cm diameter grooves. The shaped doughs were then proofed in a climatic chamber (Tecnomac Lev2+, Castel MAC Srl., Veneto, Italy) at a temperature of 30 °C and a relative humidity (RH) of 75%. The leavening process was completed when the initial volume had doubled, which took about 35 min. After baking for 16 min in an electric oven (Europa, Malo, VI, Italy) at 200 °C, the breadsticks were cooled for 30 min before the analysis. Three batches for each sample were made.

Sample codes were assigned to the doughs according to the type of extract used (BE, LE, or SE) and the level of supplementation (1 or 2): DCTRL (control dough sample), DBE 1, DBE 2, DLE 1, DLE 2, DSE 1, and DSE 2. To distinguish breadstick samples from dough samples, codes beginning with B were assigned: BCTRL for the control breadstick sample and BBE 1, BBE 2, BLE 1, BLE 2, BSE 1, and BSE 2 for the fortified breadsticks.

### 2.5. Determination of Phenolic Fractions of Doughs and Breadsticks

The methodologies previously elucidated by Conte et al. (2021) were employed to determine the soluble and insoluble phenolic fractions in dough and breadstick samples [7]. In detail, the soluble fraction was extracted twice from 1 g of finely ground sample using 2 mL of a HCl conc/methanol/water (1:80:10, *v*/*v*, Carlo Erba Reagents, Ltd., Milan, Italy) mixture under agitation for two hours at room temperature. After filtering and recovering the supernatants, the sample residues were subjected to extraction of hydrolyzable (insoluble) polyphenols with 5 mL of a methanol/concentrated sulfuric acid solution (10/1, *v*/*v*, Carlo Erba Reagents, Ltd., Milan, Italy), for twenty hours in a shaking water bath at 85 °C. The extracts were then made to react with Folin–Ciocalteau reagent (Carlo Erba Reagents, Ltd., Milan, Italy) and a 7.5% sodium carbonate solution (Carlo Erba Reagents, Ltd., Milan, Italy) and analyzed using a spectrophotometer at a wavelength of 750 nm. The analyses were repeated twice, and the results were expressed in mg of gallic acid (Carlo Erba Reagents, Ltd., Milan, Italy) equivalent (GAE) per 100 g of dry matter (d.m.) through calibration curves. The total polyphenol content was calculated by the sum of soluble and insoluble fractions.

### 2.6. Dough Rheological Measurements

#### 2.6.1. Dough Extensibility (Kieffer Test)

As previously reported by Dahdah et al. (2024), the uniaxial extensional properties were assessed with a texture analyzer (TA-XT2i, Stable Micro System, Surrey, UK) equipped with the Kieffer extensibility rig (A/KIE, Stable Micro Systems, Surrey, UK) and a 30 kg load cell [18]. A small portion of dough (30 g) was gently manipulated into a cylindrical shape, placed in a Teflon mold (which had been sprinkled with paraffin oil to prevent sample adhesion) and pressed with a clamp to gain uniform dough strips. The excess dough was then removed with a spatula. The strips (still inside the press) were kept at 25 °C and 75% RH in a climate chamber for 40 min to allow relaxation of the dough structure. Next, the tensile test was conducted on 6 dough strips per batch taken from the center of the mold, at the following conditions: pre-test speed 2.0 mm/s, trigger force 5 g, test speed 3.3 mm/s, and post-test speed 10.0 mm/s. At the end of the test, a force–distance curve was generated by Texture Exponent TEE32 software (v. 6.1.10.0 Stable Micro System, Surrey, UK), from which the following parameters were determined: resistance of extension (the maximum peak force recorded), expressed in N, and extensibility expressed in mm (the distance needed to break the dough strips).

#### 2.6.2. Dough Stickiness

The measurement of dough stickiness was performed using the SMS/Chen-Hoseney dough stickiness rig (A/DSC) and a 25 mm Perspex cylinder probe (P/25P) (Stable Micro-Systems, Surrey, UK) attached to the texture analyzer. A small quantity of dough was placed within the sample chamber of the kit. After removing the excess sample, a 1 mm high portion of the sample was extruded through the holes of the lid by turning the screw inside the cell. This was then promptly covered with the Perspex cap to minimize moisture loss. After a 30 s rest, the chamber was placed under the cylinder probe for analysis. The dough was then removed with a spatula and extruded again, repeating the test six times per batch. Stickiness values were derived as peak positive maximum force, expressed in N, from the force–time graph generated during the test by the Texture Exponent TEE32 software (v. 6.1.10.0 Stable Micro System, Surrey, UK).

### 2.7. Breadstick Measurements

#### 2.7.1. Texture Analysis

A three-point bending test was used to evaluate the textural properties of 30 breadstick halves for each sample (BC, BBE 1, BBE 2, BLE 1, BLE 2, BSE 1, and BSE 2), one hour after their preparation, in accordance with the methodology previously outlined by Conte et al. (2021) [7]. For these measurements, a texturimeter (TA-XT2 Texture Analyzer, Stable Microsystems, Surrey, UK) equipped with a 30 kg load cell and a three-point bending rig (HDP/3PB) was employed. Each sample was placed on the two moveable supports of the rig base, positioned 60 mm apart, and fractured by the probe blade, which was slid downward at a pre-test speed of 1 mm s^−1^ and a test speed of 3 mm s^−1^. The software outputs force–distance curves, from which the hardness and brittleness parameters were obtained. These parameters correspond to the maximum force required to snap the sample (N) and the distance travelled by the blade before the breadstick cracked (mm), respectively.

#### 2.7.2. Moisture Content and Water Activity

Moisture content and water activity (a_w_) measurements were performed on the ground breadsticks with a moisture analyzer set at 105 °C with a standard heating profile (KERN & SOHN GmbH, Model Kern-DAB 100-3, Balingen, Germany) and an electronic hygrometer (Rotronic, Model Aw-Win, Bassersdorf, Switzerland) paired with a Karl-Fast probe, respectively. The analyses were replicated five times.

#### 2.7.3. Color Determination

Color parameters (lightness L*, redness–greenness a*, and yellowness–blueness b*) were measured on the freshly ground sample to prevent measurement inaccuracies due to the small caliber of the breadsticks. Ten measurements were made on each sample using the tristimulus colorimeter (Minolta CR-300, Konica Minolta Sensing, Osaka, Japan) equipped with a measuring head CR-300 and granular material equipment CR-A50. Additionally, the total color difference (Δ*E*) was calculated using the equation below:

(1) $$∆E={\left(\left({∆L}^{2}\right)+\left({∆a}^{2}\right)+\left({∆b}^{2}\right)\right)}^{1/2}$$

#### 2.7.4. Antioxidant Capacity

The antioxidant capacity of the breadsticks was determined using the DPPH and ABTS spectrophotometric assays. Briefly, 3 g of finely ground sample was subjected to extraction with 10 mL of a methanol–water solution (50:50 *v*/*v*, Carlo Erba Reagents, Ltd., Milan, Italy) acidified with hydrochloric acid (pH 2) (Carlo Erba Reagents, Ltd., Milan, Italy) for 1 h at room temperature and under constant stirring. After centrifugation (3500 rpm, 10 min) of the sample and collection of the supernatant, the residue was extracted a second time under the same conditions, but with 10 mL of an acetone–water solution (70:30 *v*/*v*, Carlo Erba Reagents, Ltd., Milan, Italy). The supernatant obtained from the second extraction was mixed with the previous one and used for the determination of antioxidant capacity, performed with the two spectrophotometric assays (DPPH and ABTS) used for by-product extracts, as explained in the paragraph above (2.3). The antioxidant capacity, conducted in duplicate, was expressed as mg of Trolox (Sigma-Aldrich, Milan, Italy) equivalent (TE) per g of d.m.

#### 2.7.5. Oxidation Stability (OXITEST)

The oxidation stability of the breadstick samples was evaluated using the OXITEST reactor (VELP Scientifica, Usmate Velate, MB, Italy), according to the AOCS International Standard Procedure [19], which speeds up lipid oxidation reactions through two accelerating factors, temperature and oxygen pressure. Specifically, 30 g of homogeneous, finely ground sample (10 g per titanium sample holder) was placed inside each reaction chamber of the instrument. The samples were then exposed to an oxygen pressure of 6 bar at three different temperatures (80, 90, and 100 °C). All measurements were performed in duplicate. At the end of the tests, the induction period (IP) at the specific temperature was automatically calculated from the pressure–time curves obtained by the dedicated OXISoft^TM^ 3.0.0 software when the flex was reached. In fact, the bending curve corresponds to the end of the product’s intrinsic resistance to lipid oxidation and the beginning of accelerated oxygen adsorption. After evaluating the repeatability of the IP data for each sample and its linear dependence on temperature, the software calculated a linear regression equation on a semi-logarithmic scale (log of the IP–temperature curve). This equation was used to estimate the shelf-life of the products at the specified storage temperature (22 °C) [7]. The results were expressed in days, and correlation coefficients (R^2^) were reported.

### 2.8. Statistical Analysis

The experimental data were subjected to one-way analysis of variance (ANOVA) followed by Fisher’s least significant difference (LSD) test to separate means with a 95% confidence interval. The t-test was used to evaluate the differences between the doughs and the resulting breadsticks in order to assess the effect of baking on the content of total polyphenols and their respective fractions. Pearson correlation analysis was also employed to investigate the relationships among the analyzed parameters. Statistical analyses were performed using Statistica 12.0 software (StatSoft, Inc., Tulsa, OK, USA).

## 3. Results and Discussion

### 3.1. Total Polyphenol Content and Antioxidant Capacity of By-Product Extracts

Table 1 presents the findings of the total polyphenol content and antioxidant capacity of the extracts derived from artichoke by-products (BE, LE, and SE). As illustrated in the table, the SE exhibited a significantly (*p* < 0.05) higher polyphenol content than the BE and the LE samples. The data obtained from the two antioxidant capacity assays reflected the findings of the total polyphenol content, with the SE exhibiting the highest antioxidant capacity in both assays. Furthermore, the BE sample demonstrated a significantly higher antioxidant capacity than the LE sample, particularly in the case of ABTS.

The results obtained were found to be lower than those previously reported for the three Spinoso sardo by-product fractions gathered during a distinct harvesting season [10]. It is known that the climatic and edaphic environment, in addition to the management of the crop, exerts a considerable influence on the content of antioxidants, particularly polyphenols, which are affected by both genetic factors and external variables [20]. Indeed, there are polyphenols that are normally synthesized by the plant during the development of plant tissues and are species-specific, while others are produced in response to biotic or abiotic stresses [21].

### 3.2. Rheological Parameters of Dough Samples (Dough Stickiness and Extensibility)

The rheological properties of a dough play a crucial role during processing steps after kneading, in particular, in the sheeting and molding operations. A proper balance of viscoelastic properties is required. If the elastic component is too dominant, the dough springs back too far after sheeting, and it becomes difficult to give it the desired final shape; on the other hand, a too extensible dough is also undesirable for the molding phase. Furthermore, a dough that is excessively sticky can lead to major issues, resulting in significant downtime on the production line. In order to evaluate the effect of the introduction of polyphenol-rich extracts obtained from artichoke by-products on dough technological properties, the parameters of dough stickiness, extensibility, and resistance to extension were analyzed, and the results are summarized in Table 2.

The data revealed that the incorporation of the artichoke by-product extracts at both levels of addition did not significantly (*p* > 0.05) affect the rheological parameters of resistance to extension and extensibility. Conversely, significant differences (*p* < 0.05) were observed in the stickiness parameter, whereby only the doughs enriched with LE differed from all other samples, displaying the higher values. Although no significant differences were found in the other samples, there was a tendency for the extracts to increase the stickiness of the dough. It is known that gluten proteins interact through disulfide bonds, hydrophobic cross-links, and hydrogen bonds, providing the basis for network formation. However, there are factors that can affect this structure, such as phenolic compounds, which not only improve the nutritional profile of dough but also inhibit gluten disulfide cross-linking. As a result, their incorporation results in specific rheological alterations [22,23]. In fact, several authors have demonstrated that different classes of phenolic compounds can differentially influence the texture of fortified doughs. For instance, phenolic acids have been documented to reduce dough mixing strength and lead to the formation of sticky doughs [24]. The incorporation of caffeic and ferulic acids, quercetin, and black rice anthocyanins resulted in a decrease in dough stability and resistance to extension [22,25,26,27]. Conversely, the inclusion of tannic acids and oleuropein improved the properties of the doughs, making them stronger, more elastic, and less sticky [28,29]. Moreover, the presence of flavonoids (in both aglycone and glycoside forms) influenced gliadin conformation, particularly affecting disulfide bridges [30]. Indeed, these proteins are involved in the adhesive behavior of dough and are known to enhance the stickiness of dough [31,32].

It can thus be postulated that the significant increase in dough stickiness observed in the present study may be attributed to the different phenolic composition of the leaf extracts, in comparison to those obtained from bracts and stems. This hypothesis is corroborated by the findings of a previous study conducted by the same authors, in which it was observed that the leaf extracts had a higher concentration of flavonoids, including luteolin 7-*O*-glucoside and apigenin 7-*O*-rutinoside, than extracts from bracts and stems [10].

### 3.3. Moisture and Water Activity of Breadstick Samples

As can be seen in Table 3, a comparison of the control and fortified breadsticks revealed differences that were not always statistically significant with regard to both moisture and a_w_ parameters. The moisture values measured on the fortified breadsticks ranged from 10.63% of the BLE 2 sample to 13.27% of the BBE 2, compared to 9.66% for the BCTRL. These results fall within the wide range reported in the literature for moisture content for breadsticks (mean values: 6.63–15.52%) [33,34]. In general, the addition of the extracts resulted in a slight increase in the final moisture content of the fortified breadsticks. Particularly with the addition of bract extract (BE), this increase was significant (*p* < 0.05) with respect to the control and became more pronounced with higher addition levels. This was probably due to the presence of fiber in the extracts used for the supplementation, which could affect the absorption capacity of the resulting breadsticks. In fact, artichoke by-products, especially bracts, are a potential source of dietary fiber, mainly pectin and inulin [35,36]. In addition, ethanol–water mixtures are not only efficient for the extraction of polyphenols but can also allow the recovery of moderate amounts of inulin from lyophilized artichoke by-products [37,38,39]; furthermore, the use of ultrasound can facilitate the extraction of pectin and other saccharides, through the disruption of cell walls by the phenomenon of cavitation [40,41].

A comparable trend was identified for the a_w_ parameter, which demonstrated an increase in line with the moisture content. The BCTRL recorded a value of 0.56, while in the fortified breadsticks, the values exhibited a range from a maximum of 0.73 in BBE 2 to a minimum of 0.61 in BLE 2. As in the case of the moisture parameter, the addition of BE caused a significant (*p* < 0.05) increase in the available water in the finished products compared to the control, especially at the high level of supplementation. In support of this, the positive value of the correlation coefficient (r) showed that a higher moisture content corresponded to a greater a_w_ value (r = 0.971; *p* < 0.001).

### 3.4. Textural and Color Parameters of Breadstick Samples

Two of the most influential factors affecting consumer acceptance are the texture and color of the finished baked products. In this context, the values of hardness, brittleness, and colorimetric coordinates (L*, a*, and b*) of the breadsticks were analyzed and reported in Table 4.

With regard to texture, a tendency towards a decrease in hardness was observed in comparison to the control sample (40.90 ± 0.94 N) when the extracts were added. However, only the samples prepared with BE exhibited a statistically significant difference (*p* < 0.05). Furthermore, the fortified breadsticks, with the exception of BLE 1, had significantly higher brittleness values, resulting in greater deformation before breaking than the BCTRL sample, which exhibited a crumblier structure with a value of 1.02 ± 0.01 mm. The most pronounced increase was measured in the BBE 2 sample (1.87 ± 0.17 mm). The observed differences in texture parameters were probably related to the higher moisture content of the supplemented breadsticks, particularly those prepared with bract extracts, in comparison with the control. In fact, there is evidence that an increase in moisture content in foods with a rigid and brittle structure results in an enhanced rubbery and flexible behavior [42]. A similar mechanical behavior was observed in gluten-free breadsticks fortified with phenolic-rich extracts obtained from olive oil by-products [7]. The results obtained were also corroborated by the significant inverse correlation between moisture and hardness parameters (r = −0.822; *p* < 0.001) and the significant positive correlation between the values of moisture and brittleness (r = 0.806; *p* < 0.001).

With regard to color, the incorporation of the extracts had a significant (*p* < 0.05) effect on the colorimetric parameters, as can be seen in Table 4. In particular, a general reduction in the a* colorimetric coordinate with respect to the control was observed in all the fortified samples, with the exception of BSE 1. This decrease was more pronounced in the samples obtained by adding BE, which exhibited negative values, indicating a tendency towards green especially at higher addition levels. This was probably due to the bright green color of the bract extract. The color of BE also affected the b* coordinate. Indeed, the samples BBE 1 and BBE 2 exhibited the lowest values, denoting a reduced yellow tendency, in comparison to the other samples. Conversely, as the color of the stem extract tends towards yellow ochre, among fortified breadsticks, those prepared with SE exhibited the highest values of the a* and b* parameters, resulting in more reddish and yellowish tones. However, these color differences, as also evident from the measured values of ΔE, were not readily evident to the human eye, suggesting that the extracts exerted only a minimal influence on the color of the resulting breadsticks. Indeed, as previously documented [43], according to the criteria established by the International Commission on Illumination, values of ΔE within the range of 0–2.0 indicate an unrecognizable color difference. Values between 2.0 and 3.5 indicate differences that are recognizable even by an inexperienced observer. Finally, values above 3.5 indicate differences that are obvious to the human eye.

### 3.5. Polyphenol Content of Dough and Breadstick Samples

Typically, fruit and vegetables contain a significant proportion of phenolics in a soluble form, whereas cereals serve as an excellent source of insoluble-bound polyphenols [44]. Therefore, to more accurately assess the composition of phenolic compounds in the fortified breadsticks, both fractions of polyphenols—soluble (SF) and insoluble (IF)—were determined. Moreover, due to the inherent instability and reactive nature of these compounds, as well as the inevitable degradation that occurs as a result of heat and oxidation during the baking phase, a preliminary analysis was conducted on the doughs with the aim of assessing the impact of the thermal process on the SF and IF content.

In the present study, a comparison of the DCTRL sample with the extract-fortified doughs revealed significant differences (*p* < 0.05) in the total polyphenol content with the exception of the DBE 1 and DLE 1 samples (Table 5). In particular, the highest values were recorded in the DSE 2 and DBE 2 samples, closely followed by the DSE 1 and DLE 2 doughs. The greater contribution of SE and BE can be justified by their higher polyphenol content in comparison to LE (see Section 3.1). A more detailed examination of the data revealed that the addition of artichoke extracts mainly affected the SF of the resulting doughs, with a more pronounced impact at higher addition levels of SE and BE (98.0 ± 7.0 mg of GAE 100 g^−1^ and 95.6 ± 5.9 mg of GAE 100 g^−1^, respectively). In contrast, no significant change in the concentration of SF was observed in the doughs obtained when LE was added at both levels, in comparison with the control. The IF exhibited minimal alteration, with a slight, though not statistically significant, tendency to increase observed in nearly all fortified doughs when compared to the control. The sole exception was the DLE 1 sample, which exhibited the lowest IF concentration (mean value: 155.0 ± 4.4 mg of GAE 100 g^−1^). The limited impact of the extract addition on the IF concentration observed in all doughs is presumably attributable to the low proportion of phenolic compounds present in an insoluble form in the fresh artichoke (1.81–3.11% of the total amount) [45]. Moreover, the food-grade solvents used to obtain the extracts may have enabled the predominant extraction of polyphenols in soluble form over those in insoluble form, which require a more rigorous extraction procedure.

After baking, all the breadstick samples exhibited similar levels of polyphenol content with values ranging from 244 to 248 mg of GAE 100 g^−1^ (Table 5).

However, a more detailed examination of the data evidenced that the baking process exerted a different influence on soluble and insoluble polyphenols. With regard to the SF, a significant reduction was observed in all samples with respect to the dough samples, which can be attributed to the lower stability and higher susceptibility to high temperatures than the IF [46]. Notably, the highest decrease, amounting to approximately 37%, was observed in breadsticks fortified with LE at both levels of addition. In contrast, fortification with SE, despite a baking loss ranging from 31% to 35%, yielded the highest SF concentration even after baking (61.6 ± 1.3 mg of GAE 100 g^−1^ in BSE 1 and 64.1 ± 0.6 mg of GAE 100 g^−1^ in BSE 2). No significant differences were observed between the two samples fortified with BE and the BCTRL.

With regard to the IF, all finished products, with the exception of BBE 2, exhibited a significant increased concentration following the baking process (in comparison to the dough samples), although no significant differences were observed within the breadstick samples. This is probably due to the formation of heat-induced compounds resulting from the Maillard reaction [47]. It is important to highlight that the samples prepared with the incorporation of LE, despite undergoing a more pronounced decline in the SF, also exhibited the most notable increase in the IF. This may be indicative of a reallocation of compounds within the sample, rather than a loss through degradation. Domínguez-Fernández et al. (2021), by studying the effects of different cooking techniques on the phenolic profile of artichokes, found that these compounds may undergo degradation but also redistribution due to isomerization and hydrolysis reactions [45].

In summary, the significant differences in total polyphenol content observed in the dough samples were no longer detectable in the breadsticks due to the distinct influence exerted by the baking process on the soluble and insoluble fractions of the samples.

### 3.6. Antioxidant Capacity of Breadstick Samples

The estimation of the antioxidant capacity of the breadsticks using the two methods gave comparable results, as evidenced by Pearson’s analysis, which demonstrated a strong positive and significant correlation (r = 0.945, *p* < 0.001) between DPPH and ABTS values. As illustrated in Table 6, an upward significant trend was observed with the addition of increasing quantities of the extracts with respect to the control, with the exception of BLE 1 and BLE 2 samples (*p* < 0.05). In particular, the significantly higher results were recorded in both spectrophotometric assays in the samples fortified with 2000 ppm of the extracts of both the stem (with increases of + 69% for DPPH and +26% for ABTS compared to the BCTRL) and the bract (with increments of +57% for DPPH and +33% for ABTS), immediately followed by the samples prepared with the lowest level of the same extracts. Similar increments in the antioxidant capacity were obtained in salted baked snacks fortified with the addition of olive leaf extract. However, the authors employed a lower fortification level (400 ppm), which may be attributed to the initial higher polyphenol concentration of the olive industry by-product [17].

The greater efficacy of SE and BE in increasing the antioxidant power of breadsticks appeared to diverge from the findings observed in the total polyphenol content, where no significant differences among the samples were found. It is established that the antioxidant capacity is influenced not only by the total amount of phenolic compounds present, but also by their composition. Indeed, this appears to be positively influenced by the number of hydroxyl groups and their position on the aromatic ring, particularly on the ortho or para position [48]. In full agreement with this observation, a study conducted by Wang et al. (2003), by analyzing the DPPH scavenging activity of individual phenolic compounds extracted and purified from artichoke leaves and flower heads, found that compounds such as cynarin (1, 3-di-caffeoylquinic acid), luteolin rutinoside, cynaroside (luteolin 7-O-glucoside), and chlorogenic acid had higher antioxidant activity than 1-caffeoylquinic acid and apigenin 7-rutinoside [49].

As previously reported by the same authors [10], the stem and bract extracts used in the present study were found to be particularly rich in compounds, including chlorogenic acid, 1,5-di-*O*-caffeoylquinic acid, and 3,5-di-*O*-caffeoylquinic acid, which possess multiple hydroxyl groups in the ortho position. These compounds were either absent or present in low concentrations in the leaf extract. It can thus be concluded that the enhanced antioxidant capacity observed in the SE- and BE-fortified breadsticks is likely attributable to the higher concentration of the caffeoylquinic acid derivatives present in these extracts.

### 3.7. Estimated Shelf-Life of Breadsticks with OXITEST

A variety of factors related to composition and nutritional properties, as well as packaging and storage conditions, can influence the shelf-life of food products. In the case of baked goods such as breadsticks, which often require significant amounts of fat to achieve the desired texture and flavor, lipid oxidation can play a crucial role in defining their shelf-life [16].

Indeed, lipids undergo oxidative degradation as a consequence of complex chemical chain reactions that involve fatty acids and oxygen. This degradation phenomenon is also referred to as rancidity, as it results in the formation of intermediate compounds (free radicals) and secondary compounds (such as aldehydes, ketones, and hydrocarbons) that contribute to the development of undesirable flavors, which can negatively impact the quality of the food product. The rate of lipid oxidation is influenced by different factors, including the fatty acid composition, the storage conditions (e.g., temperature, light, oxygen availability, and water activity), and the presence of pro-oxidants and antioxidants [50]. Plant extracts, for instance, are known to contain a multitude of natural antioxidants, including phenolic acids, flavonoids, and anthocyanins. These antioxidants act as free-radical scavengers by virtue of their multiple hydroxyl groups, which function as hydrogen donors, preventing the reaction of peroxyl or alkoxy radicals with new fatty acids [50]. Therefore, the use of artichoke by-product extracts may represent an effective strategy for the enhancement of the oxidative stability of breadsticks and consequently their shelf-life.

The accelerated oxidation tests, conducted at three different temperatures (80, 90, and 100 °C) and a constant overpressure (6 bar) in the OXITEST reactor, revealed a linear relationship between the IPs and temperatures, as evidenced by the R^2^ values greater than 0.99 (Table 7). Indeed, an overall decrease in the IP was noted as operating temperature increased across all samples. This allowed for the estimation of the shelf-life of the breadsticks at a temperature of 22 °C.

In general, the lowest lipid stability to oxidation was observed in the BCTRL, which showed IPs of approximately 22 and 4 h at 80 and 100 °C, respectively and an estimated shelf-life of 109 ± 1 days. The incorporation of the extracts, especially at the highest levels, allowed an improvement in the oxidative stability of the resulting breadsticks. In line with the data of antioxidant capacity, the addition of 2000 ppm of SE, BE, and LE ensured a significant (*p* < 0.05) extension of the shelf-life of breadsticks by 62, 44, and 29%, respectively. The BLE 1 and BBE 1 samples did not show a significant increment with respect to the control. In contrast, the extract obtained from the stems, even at the lowest level of addition (1000 ppm), caused an improvement in shelf-life of almost twice as much (+44%). Similarly, Hammad et al. (2021) recorded a significant increase in the oxidative stability of breadsticks fortified with ginseng dried extract, with an extension recorded of up to 55 days at room temperature [8]. Conversely, the incorporation of grape pomace was observed to have a deleterious effect on the OXITEST estimated shelf-life of breadsticks, resulting in an accelerated oxidation rate. However, in this case, the fortification was conducted with the by-product in powder form, which likely also contained pro-oxidant molecules or polyunsaturated fatty acids [6].

The highly significant (*p* < 0.01) positive correlations between antioxidant capacity and estimated shelf-life (r = 0.787 and r = 0.710 with DPPH and ABTS data, respectively) found in the present study substantiate the close association between these two parameters.

## 4. Conclusions

The findings of the present study indicate that the use of hydroalcoholic extracts derived from various by-products of the artichoke industry to enhance the antioxidant activity and extend the shelf-life of globally consumed snacks such as breadsticks represents a promising strategy for reducing both the environmental and economic footprint of the artichoke industry. This is the first demonstration of the capacity of these polyphenol-rich hydroalcoholic extracts to enhance oxidative stability without prior stabilization. This also offers a potential solution to reduce the impact of food waste and loss of a valuable economic resource within the Mediterranean agricultural sector, to promote a circular economy, and to increase the competitiveness of the artichoke industry and of snack manufacturers. Furthermore, the exhausted by-product residues, which could be considered as lignocellulosic biomass, may have the potential to be repurposed as a sustainable source for bioethanol production or in the textile industry, thereby reinforcing the concept of circularity. Ultimately, this could result in a finished product with higher selling prices and a reduction in waste at the consumer level. Indeed, despite a slight decline in texture observed in the fortified breadsticks, the incorporation of extracts, particularly at the highest levels of SE and BE, demonstrated the capacity to enhance antioxidant activity and extend the shelf-life, without discernible alterations in the snack’s final color. Additionally, the rheological data of the dough indicated that the incorporation of the extracts did not affect the dough’s workability, with the exception of the LE, which significantly increased its stickiness. Of the extracts evaluated, the one derived from artichoke stems exhibited the most promising results as a natural preservative and nutritional improver. However, given the high sensitivity of artichoke phenolic compounds, a stabilization intervention such as encapsulation would probably yield better results. Therefore, this aspect should be examined in depth to ascertain the effectiveness of the treatment. Moreover, additional research is necessary to ensure the reliability and consistency of the existing findings, particularly in light of the inherent variability in the phenolic content of artichoke by-products, which is strongly influenced by soil and climatic conditions. Further investigations are required to assess the impact of these hydroalcoholic extracts on the economic aspect through a life cycle assessment, and on the sensorial properties of the finished product.

## Figures and Tables

**Table 1:** Total polyphenol content and antioxidant capacity of artichoke by-product extracts.

Sample ^1^	Total Polyphenols mg GA 100 g^−1^ d.m.	DPPH TE μmol TE g^−1^ d.m.	ABTS μmol TE g^−1^ d.m.
BE	1539 ± 64 b	39.77 ± 2.30 b	62.18 ± 5.19 b
LE	1256 ± 18 c	32.37 ± 0.60 b	55.05 ± 3.81 c
SE	2163 ± 206 a	86.49 ± 16.31 a	90.19 ± 9.20 a

^1^ Mean value ± f deviation. Different letters in the same column denote significant differences (*p* < 0.05) according to the least significant difference (LSD) test; BE: bract extract; LE: leaf extract; SE: stem extract.

**Table 2:** Rheological parameters of control and fortified doughs.

Samples ^1^	Resistance to Extension (N)	Extensibility (mm)	Stickiness (N)
DCTRL	0.13 ± 0.02 a	63.44 ± 0.02 a	0.35 ± 0.02 b
DBE 1	0.12 ± 0.00 a	63.29 ± 0.04 a	0.35 ± 0.00 b
DBE 2	0.12 ± 0.02 a	62.51 ± 3.07 a	0.39 ± 0.06 b
DLE 1	0.12 ± 0.02 a	60.27 ± 1.40 a	0.42 ± 0.04 a
DLE 2	0.11 ± 0.02 a	63.60 ± 1.03 a	0.45 ± 0.04 a
DSE 1	0.11 ± 0.05 a	61.55 ± 1.28 a	0.37 ± 0.03 b
DSE 2	0.11 ± 0.02 a	61.47 ± 3.34 a	0.40 ± 0.01 b

^1^ Mean value ± standard deviation. Different letters in the same column denote significant differences (*p* < 0.05) according to the least significant difference (LSD) test; DCTRL: control dough; DBE 1: dough with 1000 ppm of bract extract; DBE 2: dough with 2000 ppm of bract extract; DLE 1 and 2: dough with 1000 and 2000 ppm leaf extract, respectively; DSE 1 and 2: dough with 1000 and 2000 ppm stem extract, respectively.

**Table 3:** Moisture content and water activity (a_w_) of control and fortified breadsticks.

Samples ^1^	Moisture Content %	a_w_
BCTRL	9.66 ± 0.11 d	0.56 ± 0.01 d
BBE 1	13.03 ± 0.61 ab	0.73 ± 0.01 ab
BBE 2	13.27 ± 0.20 a	0.73 ± 0.01 a
BLE 1	11.36 ± 0.29 bcd	0.63 ± 0.04 bcd
BLE 2	10.63 ± 1.84 cd	0.61 ± 0.08 cd
BSE 1	11.49 ± 0.82 abcd	0.66 ± 0.04 abcd
BSE 2	11.83 ± 0.08 abc	0.69 ± 0.03 abc

^1^ Mean value ± standard deviation. Different letters in the same column denote significant differences (*p* < 0.05) according to the least significant difference (LSD) test; BCTRL: control; BBE 1: breadsticks with 1000 ppm of bract extract; BBE 2: breadsticks with 2000 ppm of bract extract; BLE 1 and 2: breadsticks with 1000 and 2000 ppm leaf extract, respectively; BSE 1 and 2: breadsticks with 1000 and 2000 ppm stem extract, respectively.

**Table 4:** Textural and color properties of control and fortified breadsticks.

Samples ^1^	Hardness (N)	Brittleness (mm)	L*	a*	b*	Δ*E*
BCTRL	40.90 ± 0.94 a	1.02 ± 0.01 d	62.48 ± 0.39 c	0.87 ± 0.17 a	17.72 ± 0.36 bc	-
BBE 1	35.75 ± 2.66 bc	1.68 ± 0.24 ab	62.93 ± 0.14 b	−0.15 ± 0.02 e	16.62 ± 0.17 e	1.56
BBE 2	31.75 ± 2.00 c	1.87 ± 0.17 a	63.53 ± 0.36 a	−0.39 ± 0.06 f	17.01 ± 0.30 e	1.79
BLE 1	39.14 ± 1.47 ab	1.32 ± 0.05 cd	63.31 ± 0.07 a	0.31 ± 0.09 d	17.02 ± 0.44 d	1.22
BLE 2	38.90 ± 0.34 ab	1.43 ± 0.19 bc	62.65 ± 0.64 bc	0.43 ± 0.12 c	17.52 ± 0.30 c	0.51
BSE 1	38.21 ± 2.23 ab	1.61 ± 0.00 abc	62.06 ± 0.46 d	0.85 ± 0.07 a	18.54 ± 0.38 a	0.91
BSE 2	36.97 ± 1.02 ab	1.46 ± 0.15 bc	62.68 ± 0.19 bc	0.62 ± 0.08 b	17.96 ± 0.50 b	0.40

^1^ Mean value ± standard deviation. Different letters in the same column denote significant differences (*p* < 0.05) according to the least significant difference (LSD) test; BCTRL: control; BBE 1: breadsticks with 1000 ppm of bract extract; BBE 2: breadsticks with 2000 ppm of bract extract; BLE 1 and 2: breadsticks with 1000 and 2000 ppm leaf extract, respectively; BSE 1 and 2: breadsticks with 1000 and 2000 ppm stem extract, respectively.

**Table 5:** Total and polyphenol fraction content of dough and breadstick samples.

Samples ^1^	Soluble Fraction mg GAE 100 g^−1^ d.m.	Insoluble Fraction mg GAE 100 g^−1^ d.m.	Total Polyphenol Content mg GAE 100 g^−1^ d.m.
Doughs			
DCTRL	83.5 ± 2.3 cA	157.7 ± 0.8 abB	241.2 ± 3.1 cA
DBE 1	86.2 ± 3.6 cA	160.1 ± 0.0 abB	246.3 ± 3.6 bcA
DBE 2	95.6 ± 5.9 abA	162.9 ± 0.3 abA	258.5 ± 5.6 aA
DLE 1	82.8 ± 0.6 cA	155.0 ± 4.4 bB	237.8 ± 3.8 cA
DLE 2	88.1 ± 1.0 bcA	163.8 ± 1.0 aB	251.9 ± 2.0 abA
DSE 1	89.9 ± 0.7 abcA	163.1 ± 0.8 aB	253.0 ± 0.1 abA
DSE 2	98.0 ± 7.0 aA	162.0 ± 0.6 aB	260.0 ± 6.5 aA
Breadsticks			
BCTRL	60.4 ± 0.0 bcB	188.0 ± 8.5 aA	248.4 ± 8.5 aA
BBE 1	59.6 ± 1.6 bcB	184.3 ± 6.1 aA	243.9 ± 4.5 aA
BBE 2	61.6 ± 4.5 bcB	186.6 ± 9.7 aA	248.2 ± 5.2 aA
BLE 1	52.4 ± 1.5 dB	194.1 ± 0.3 aA	246.5 ± 1.7 aA
BLE 2	55.9 ± 0.1 cdB	192.4 ± 4.4 aA	248.3 ± 4.4 aA
BSE 1	61.6 ± 1.3 aB	186.7 ± 4.06 aA	248.4 ± 2.7 aA
BSE 2	64.1 ± 0.6 aB	181.9 ± 1.29 aA	245.9 ± 0.7 aA

^1^ Mean value ± standard deviation. Different letters in the same column denote significant differences (*p* < 0.05) within the dough samples and within the breadstick samples (lowercase letters) according to the least significant difference (LSD) test and between the doughs and the resulting breadsticks (uppercase letters) according to the *t*-test; DCTRL: control dough; DBE 1: dough with 1000 ppm of bract extract; DBE 2: dough with 2000 ppm of bract extract; DLE 1 and 2: dough with 1000 and 2000 ppm leaf extract, respectively; DSE 1 and 2: dough with 1000 and 2000 ppm stem extract, respectively; BCTRL: control breadsticks; BBE 1: breadsticks with 1000 ppm of bract extract; BBE 2: breadsticks with 2000 ppm of bract extract; BLE 1 and 2: breadsticks with 1000 and 2000 ppm leaf extract, respectively; BSE 1 and 2: breadsticks with 1000 and 2000 ppm stem extract, respectively.

**Table 6:** Antioxidant capacities of breadstick samples determined with DPPH and ABTS spectrophotometric assays.

Samples ^1^	DPPH TE μmol TE g^−1^ d.m.	∆ DPPH (%)	ABTS μmol TE g^−1^ d.m.	∆ABTS (%)
BCTRL	0.23 ± 0.01 d	-	2.25 ± 0.10 e	-
BBE 1	0.32 ± 0.05 bc	38	2.66 ± 0.17 bcd	18
BBE 2	0.37 ± 0.04 ab	57	2.98 ± 0.13 a	33
BLE 1	0.26 ± 0.02 cd	13	2.42 ± 0.22 de	8
BLE 2	0.29 ± 0.01 cd	22	2.49 ± 0.07 cde	11
BSE 1	0.35 ± 0.00 ab	49	2.75 ± 0.02 abc	22
BSE 2	0.39 ± 0.02 a	69	2.83 ± 0.05 ab	26

^1^ Mean value ± standard deviation. Different letters in the same column denote significant differences (*p* < 0.05) according to the least significant difference (LSD) test; BCTRL: control; BBE 1: breadsticks with 1000 ppm of bract extract; BBE 2: breadsticks with 2000 ppm of bract extract; BLE 1 and 2: breadsticks with 1000 and 2000 ppm leaf extract, respectively; BSE 1 and 2: breadsticks with 1000 and 2000 ppm stem extract, respectively.

**Table 7:** Estimated shelf-life of breadstick samples based on lipid oxidation data (expressed as days at 22 °C) measured with the OXITEST method.

Parameters	Samples ^1^						
	BCTRL	BBE 1	BBE 2	BLE 1	BLE 2	BSE 1	BSE 2
Estimated shelf-life (days)	109 ± 1 d	119 ± 3 cd	157 ± 4 ab	109 ± 3 d	141 ± 8 bc	156 ± 10 ab	177 ± 5 a
R^2^	0.995	0.997	0.998	0.996	0.998	0.997	0.998
Shelf-life extension (%)	−	9	44	0	29	43	62

^1^ Mean value ± standard deviation. Different letters in the same row denote significant differences (*p* < 0.05) according to the least significant difference (LSD) test; BCTRL: control; BBE 1: breadsticks with 1000 ppm of bract extract; BBE 2: breadsticks with 2000 ppm of bract extract; BLE 1 and 2: breadsticks with 1000 and 2000 ppm leaf extract, respectively; BSE 1 and 2: breadsticks with 1000 and 2000 ppm stem extract, respectively.

## Data Availability

The original contributions presented in the study are included in the article, further inquiries can be directed to the corresponding author.
